# Tissue engineering of skin and regenerative medicine for wound care

**DOI:** 10.1186/s41038-017-0103-y

**Published:** 2018-01-24

**Authors:** Steven T. Boyce, Andrea L. Lalley

**Affiliations:** 1 Department of Surgery, University of Cincinnati, P.O. Box 670558, Cincinnati, Ohio 45267-0558 USA; 2 Research Department, Shriners Hospitals for Children, Cincinnati, Ohio USA

**Keywords:** Burns, Cell therapy, Skin substitute, Tissue engineering, Wound closure, Scar, Regenerative medicine

## Abstract

Engineering of biologic skin substitutes has progressed over time from individual applications of skin cells, or biopolymer scaffolds, to combinations of cells and scaffolds for treatment, healing, and closure of acute and chronic skin wounds. Skin substitutes may be categorized into three groups: acellular scaffolds, temporary substitutes containing allogeneic skin cells, and permanent substitutes containing autologous skin cells. Combined use of acellular dermal substitutes with permanent skin substitutes containing autologous cells has been shown to provide definitive wound closure in burns involving greater than 90% of the total body surface area. These advances have contributed to reduced morbidity and mortality from both acute and chronic wounds but, to date, have failed to replace all of the structures and functions of the skin. Among the remaining deficiencies in cellular or biologic skin substitutes are hypopigmentation, absence of stable vascular and lymphatic networks, absence of hair follicles, sebaceous and sweat glands, and incomplete innervation. Correction of these deficiencies depends on regulation of biologic pathways of embryonic and fetal development to restore the full anatomy and physiology of uninjured skin. Elucidation and integration of developmental biology into future models of biologic skin substitutes promises to restore complete anatomy and physiology, and further reduce morbidity from skin wounds and scar. This article offers a review of recent advances in skin cell thrapies and discusses the future prospects in cutaneous regeneration.

## Background

Advances in burn care during the recent past have included improvements in fluid resuscitation, early wound excision, respiratory support and management of inhalation injury, improved nutrition and modulation of the hypermetabolic response, infection control and enhanced immune function, incorporation of aerobic exercise during recovery, and development of anti-scarring strategies [1]. These advances have led to significant reductions in mortality, hospital stay, and long-term morbidity. In addition to these comprehensive innovations, skin cell therapies have become part of the treatment plan for extensive burns. This review will summarize several of the most significant advances since 1980 and discuss prospects for further progress in cutaneous regeneration in the future.

## Review

### Medical needs

Cutaneous burns can generate a continuum of injuries with increasing depth into the skin. Partial-thickness burns often do not require grafting and, if debrided and treated with antimicrobial dressings, will heal spontaneously from regrowth of epithelial appendages (hair follicles, sebaceous and sweat glands) to cover the wounds. However, deep partial-thickness burns which do not heal within ~ 3 weeks and full-thickness burns require replacement of the epidermal barrier by transplantation of autologous keratinocytes. Transplantation can be accomplished by either conventional split-thickness skin grafts (STSG), applications of keratinocyte suspensions or sheets, or dermal-epidermal skin substitutes [2–5]. Autologous keratinocytes may persist indefinitely and provide permanent wound closure, whereas allogeneic keratinocytes will remain on the wound for a few days to weeks [6–8], delivering growth factors and extracellular matrix components to wounds that promote more rapid wound closure by autologous cells [9]. Combinations of widely meshed and expanded (i.e., 1:6) autografts or micrografts applied to excised, full-thickness burns and covered with allograft [10, 11] have been reported, but are slow to heal, allow granulation tissue to form, and tend to scar. Conversely, unmeshed sheet grafts applied as early as possible to critical areas (i.e., face, hands, feet, perineum) have been shown to reduce granulation tissue, minimize scar, and produce optimal functional and cosmetic outcomes [2, 12, 13].

### Biological requirements and current alternatives

Wound closure after full-thickness burns requires reestablishment of stable epidermis as a minimum requirement. Stability of the epidermis depends on reformation of the basement membrane and vascularized connective tissues to anchor the outer skin to the body. Split-thickness skin satisfies these requirements but does not replace the epidermal adnexa (hair follicles, sebaceous glands, sweat glands) or regenerate a full complement of sensory or motor nerves. Table 1 summarizes the anatomic features of uninjured skin compared to STSG, engineered skin substitutes (ESS), and healed skin after grafting. It is important to note that split-thickness skin at the first harvest does not regenerate hair follicles, sebaceous glands, or sweat glands but does contain pigmented melanocytes and vascular and neural networks which the engineered skin does not. At the second and subsequent harvests of autografts, pigmentation becomes irregular and scar is more pronounced. Compared with autografts, autologous-ESS containing cultured keratinocytes and fibroblasts may also contain “passenger melanocytes” which may colonize the wound and generate focal, but incomplete, pigmentation [14–16]. Of these deficiencies, perhaps the absence of sweat glands is most important to patients with large total body surface area (TBSA) burns because it impairs the ability to thermo-regulate properly.

**Table 1:** Comparisons of cell types in native, engineered and grafted skin (adapted from [99])

Tissue type	Cell type or structure	Uninjured skin	Split-thickness skin graft	Engineered skin substitutes	Healed skin after grafting
Epidermis	Keratinocytes	+	+	+	+
	Hair follicle	+	−	−	−
	Sebocytes	+	−	−	−
	Sweat gland	+	−	−	−
	Melanocytes	+	±	±	±
	Immune cells	+	+	−	+
	Nerve	+	+	−	±
Dermis	Fibroblasts	+	+	±	+
	Endothelial cells	+	+	−	+
	Extracellular matrix	+	±	±	±
	Smooth muscle	+	+	−	±
	Immune cells	+	+	−	+
	Nerve	+	+	−	±

Table 2 provides a partial list of acellular, temporary, and permanent skin substitutes that are either available commercially in the USA or in clinical trials. Acellular skin substitutes recruit fibro-vascular tissues from the wound bed and may consist of either biopolymers, such as collagen and chondroitin-sulfate or elastin (Integra® Dermal Regeneration Template [17]; MatriDerm®) [18], decellularized human dermis (AlloDerm™) [19], derivatized hyaluronic acid (Hyalomatrix®) [20], or polyurethane (BioTemporizing Matrix, “BTM”) [21, 22]. Each of these materials protects open wounds, promotes ingrowth of fibrovascular tissue, and may suppress granulation tissue and scar. However, the biologic materials (i.e., acellular dermis, collagen, hyaluronic acid) are prone to microbial contamination in the absence of antimicrobial agents due to their properties as biological ligands for bacteria and degradation by enzymatic activities [23, 24]. In comparison, synthetic polymers (i.e., polyurethane, poly-glycolic/poly-lactic acids) are often degraded by hydrolysis, have fewer microbial binding sites, and are less prone to microbial contamination. If used as dermal substitutes, the acellular materials may require 2 to 4 weeks to vascularize sufficiently to support a STSG. However, if used as a scaffold for cell transplantation, 2 weeks or longer for vascularization would negatively impact cell survival and reduce cellular engraftment and wound closure. Of the available acellular skin substitutes, Integra® currently has the broadest usage for extensive, life-threatening burns and burn scars in the USA [25] and has demonstrated very favorable outcomes [26, 27] since its introduction in 1996 [17]. Similar results have been described recently using the BTM material which currently remains in clinical trial [22].

**Table 2:** Biologic skin substitutes, commercially available or in clinical trial (adapted from [100])

Model [references]	Composition	Indications for use
Acellular skin substitutes		
Integra Dermal Regeneration Template [17, 101]	Bovine collagen and chondroitin-sulfate coated with silicone	Burns, reconstructive surgery
AlloDerm [19, 102]	Decellularized human dermis	Repair or replacement of damaged or inadequate integumental tissue
MatriDerm [103, 104]	Bovine collagen and elastin	Burns, reconstructive surgery
Hyalomatrix [20, 105]	Derivatized hyaluronic acid	Partial- and full-thickness wounds
BioTemporizing Matrix [22, 106]	Bilaminate degradable polyurethane	Burns, reconstructive surgery
Temporary skin substitutes (dressings)		
Cadaver allograft [29, 107]	Split-thickness skin from human donors, unfrozen or cryopreserved	Burns, reconstructive surgery
Porcine xenograft [31, 32]	Split-thickness porcine skin, cryopreserved or lyophilized	Burns, reconstructive surgery
Apligraf® [108, 109]	Allo hF in collagen gel plus stratified allo hK	Diabetic foot ulcers
StrataGraft® [33, 110]	Allo hF in collagen gel plus stratified allo hK	Partial-thickness burns
DermaGraft® [111, 112]	Allo hF on poly-galactin mesh	Diabetic foot ulcers
Permanent skin substitutes (grafts)		
EpiCel® [34, 113]	Cultured auto hK multi-layer sheet	Full-thickness burns
ReCell® [3, 114]	Uncultured suspension of auto hK, delivered as a spray	Partial-thickness burns
Reconstructed skin [36, 115]	Auto hF on acellular scaffold of dermal extracelluar matrix, plus stratified auto hK	Full-thickness burns, venous and mixed ulcers
Autologous engineered skin substitute [39, 116]	Auto hF on a collagen-GAG scaffold, plus stratified auto hK	Full-thickness burns

### Cellular skin substitutes

**Fig. 1:**
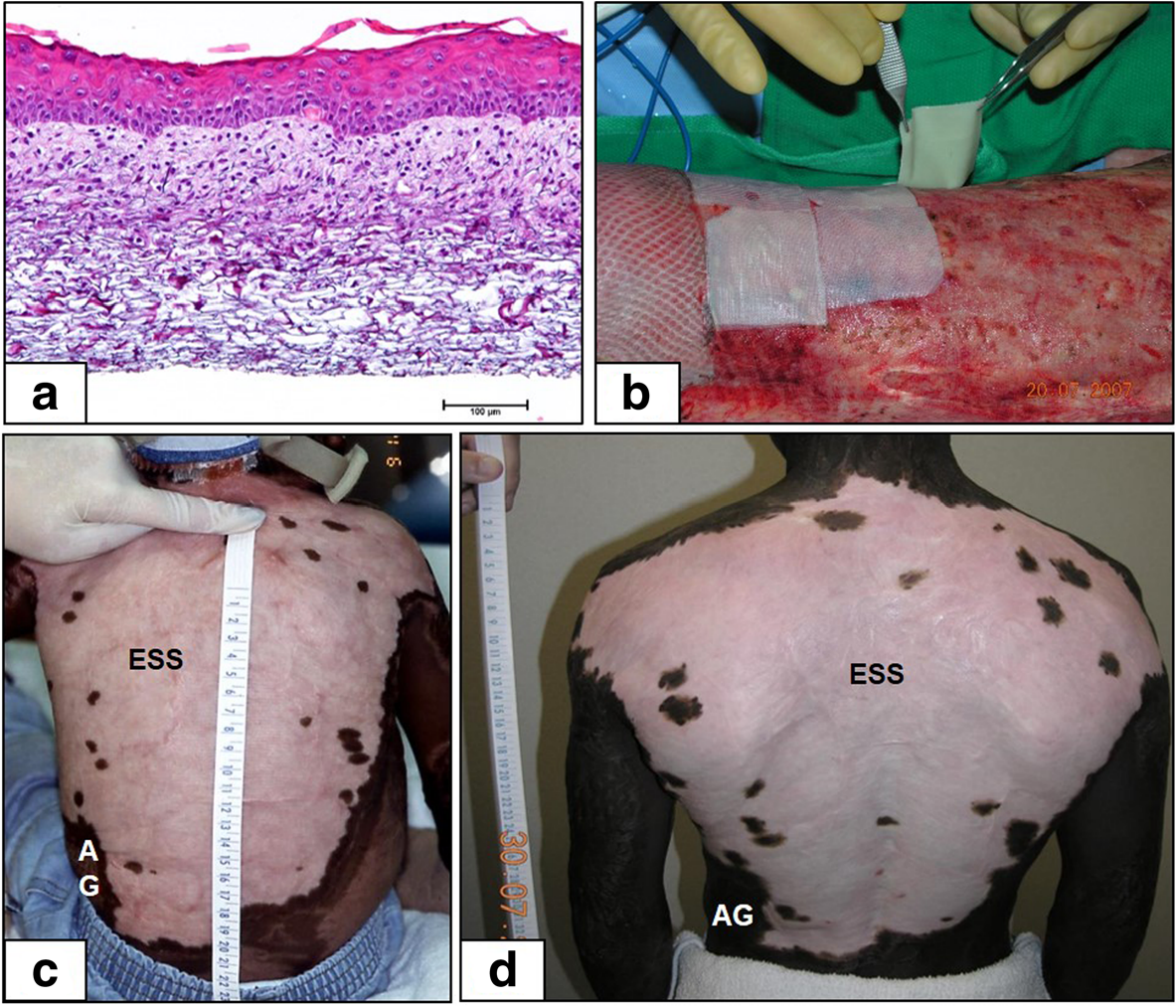
Clinical application of autologous engineered skin substitutes (ESS). **a** Histology of ESS shows a collagen-based polymer scaffold populated with cultured dermal fibroblasts and epidermal keratinocytes. Scale bar = 0.1 mm. **b** Surgical application of ESS on prepared wounds can be performed using forceps and secured with staples. **c** An African-American subject treated with ESS at 3 years of age shows predominant hypopigmentation. **d** The same subject at 14 years of age has persistent hypopigmentation but has required no reconstruction of the ESS site. Scales in centimeters

Transplantation of cellular skin substitutes has had wide-ranging results for temporary or permanent wound coverage. Temporary cellular dressings include direct harvest of split-thickness skin, available as either fresh or cryopreserved human cadaver skin [28, 29], or porcine skin with storage by chemical fixation or lyophilization [30–32]. In addition, allogeneic human fibroblasts and/or keratinocytes have been combined with degradable scaffolds (i.e., partially denatured collagen (Apligraf™; StrataGraft™) [6, 33], poly-glycolic/poly-lactic acids (DermaGraft™)) which deliver growth factors and extracellular matrix to wounds to promote autologous healing but do not persist more than a few days to weeks. Autologous keratinocytes have been applied as cultured cell sheets (EpiCel™) [34], sprayed cell suspensions prepared during surgery (ReCell™) [35], with culture-expanded fibroblasts as the dermal component [36], or in combination with a polymeric dermal scaffold populated with autologous culture-expanded fibroblasts [37, 38]. Figure 1 shows the histologic organization of a dermal-epidermal skin substitute, surgical application, and results in a pediatric patient [39]. These approaches have helped to reduce mortality in large burns (> 40% TBSA), but they lack hair follicles and glands after transplantation. Limitations of keratinocyte sheets have included poor durability and ulceration [40, 41] and with sprayed keratinocyte suspensions a requirement for co-application with widely meshed skin autograft [42] which reduces the conservation of donor skin and increases scarring after wound closure.

**Fig. 2:**
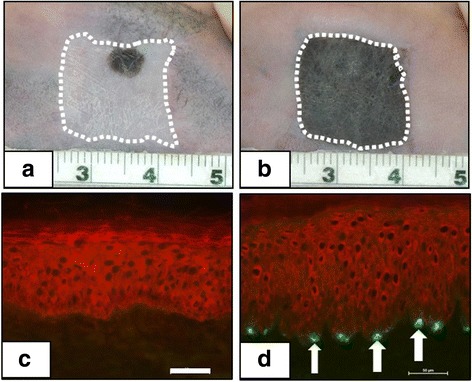
Correction of pigmentation with cultured autologous melanocytes in preclinical studies. **a** Human engineered skin substitutes (ESS) on immunodeficient mice showing hypopigmentation at 12 weeks after grafting. **b** Correction of hypopigmentation after 12 weeks by addition of isogeneic human melanocytes to ESS. Scales in centimeters. **c** Immunolabeling of epidermis with anti-cytokeratin (red) and the melanocyte-specific maker, tyrosinase-related protein-1 (TRP-1; negative). **d** Immunolabeling of ESS with added melanocytes shows epidermis (red), and TRP-1-positive melanocytes at the dermal-epidermal junction (white arrows) as in uninjured skin. Scale bars = 50 μm

**Fig. 3:**
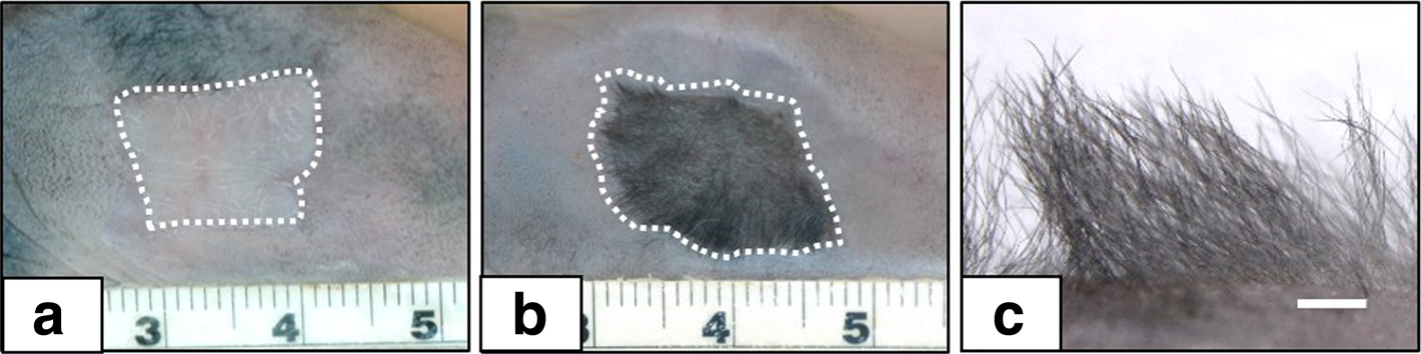
Induction of hair follicles in vivo from neonatal dermal cells grafted to immunodeficient mice. **a** Human dermal fibroblasts and human epidermal keratinocytes express no hair. **b** Neonatal murine fibroblasts and human neonatal keratinocytes express chimeric hair at 4 weeks after grafting. Scales in cm. **c** Higher magnification showing density of regenerated hair is similar to that on positive control mice. Scale = 1 mm

Preclinical investigations have reported more complex models that also include melanocytes [43–45], microvascular endothelial cells [46–48], mesenchymal stem cells [49–51], adipocyte stem cells [52], sensory nerve cells [53], hair follicle progenitor cells [54–56], or induced pluripotent stem cells (iPSCs) [57, 58]. Figure 2 shows restoration of natural skin color in human ESS with isogeneic melanocytes grafted to immunodeficient mice [59] and localization of melanocytes to their normal anatomic location at the basement membrane. These kinds of models promote activation of biological signaling pathways which may stimulate more rapid and complete healing, or drive expression of additional phenotypes to correct anatomic deficiencies. The prospective benefits of progenitor cells may include generation of additional populations of differentiated parenchymal cells (e.g., hair, sweat glands, nerve) in engineered skin grafts. Figure 3 shows the expression of hair in engineered skin containing neonatal murine skin cells [54]. As biologic complexity increases and phenotypes are restored, engineered tissues gain structures and functions that do not result from mechanisms of wound healing. These added properties may derive from embryonic or fetal mechanisms that regulate tissue morphogenesis, in addition to the mechanisms of wound healing. Together, the combination of developmental biology, wound healing, and biomedical engineering constitute the emerging field of regenerative medicine.

### Contemporary research and regenerative medicine

Although great progress has been made in reductions of morbidity and mortality in management of burn wounds, some of the most exciting advances remain ahead. These prospective advances include, but are not limited to, (a) complete restoration of skin anatomy and physiology, (b) gene therapies for specific applications, (c) automated and robotic fabrication of engineered tissues to increase efficiencies and reduce costs, and (d) quantification of wounds with non-invasive biophysical instruments.

Table 3 summarizes the anatomic and physiologic properties that may be missing from split-thickness skin autograft, ESS, or healed wounds after grafting. Among these phenotypes are epidermal barrier, dermal-epidermal junction, hair folliculogenesis and cycling, sebaceous glands, pigmentation, sensory and motor innervation, cardiovascular systems, and subcutaneous fat. Each of these phenotypes results from specific gene expression pathways that regulate its formation. Examples of these pathways are listed and referenced in the table. It is noteworthy that some of the phenotypes share regulatory pathways, such as hair follicles and sweat glands being regulated by wingless integration site of murine mammary tumor virus (Wnt), β-catenin, ectodysplasin (EDA), and its receptor (EDAR) [60, 61]. Similarly, there are members of the Sry-regulated HMG box (Sox) family of transcription factors that are expressed in formation of hair (Sox-2, -21), sebaceous glands (Sox-9), pigmentation (Sox-10), innervation (Sox-2, -10), and cardiovascular development (Sox-7, -17, -18). Despite these similarities, each pathway is expressed in a context of its microenvironment (e.g., stem cells, extracellular matrix) which also contributes to the genesis and stability of the phenotype. Undoubtedly, as continuing studies in developmental biology elucidate these pathways, greater capabilities to guide the anatomy and physiology of biologic skin substitutes will be gained.

**Table 3:** Developmental pathways and regulatory factors for cutaneous phenotypes (adapted from [100])

Cutaneous structures and phenotypes	Regulatory pathways and factors
Epidermal barrier	Ca^2+^ [117, 118] Transglutaminase, loricrin [119, 120] Essential fatty acids, stratum corneum lipids [121, 122]
Dermal-epidermal junction	Integrins [123, 124] FAK-Ras-MapK [60, 124] PKB/Akt-ERK1/2 [125]
Hair follicle genesis and cycling	Wnt/β-catenin/DKK4/BMPs [60, 61] EDA/EDAR [126, 127] Sox-2, -21 [128, 129]
Sebaceous glands	Rac1-Sox9-Lrig1 [61, 130] Wnt; Blimp1; IHH; c-myc [131] TGFβ-1 [132]
Sweat glands	Wnt/β-catenin [133] EDA/EDAR/NF-κB [134] DKK4, SHH [135]
Pigmentation	c-kit/SCF; [136] ET-3/EDNRB2 [137] Sox-10/Mitf; Eph/EphR; [138]
Sensory and motor innervation	c-jun [139] Sox-2,-10 [55] Oct-6; Krox-20; Pax 3 [140]
Cardiovascular system	Sox-7, -17, -18 [141] Mef2c/β-catenin [142] VEGF; HOXA9, VEZF1 [143]
Subcutaneous fat	PPARγ [144] Pref-1; Fabp4 [145] Myf5; Ebf2; Prdm16; Pgc-1α [146]

*EDA* ectodysplasin, *EDAR* ectodysplasin receptor, *TGFβ-1* transforming growth factor β-1, *VEGF*, vascular endothelial growth factor

Gene therapies for the skin have been studied extensively over the years and have met with limited success [62–64]. Risks from use of retrovirus-based expression systems suggest that lentiviral-mediated genetic modifications may have greater safety and efficacy in prospective studies [65, 66]. However, at least two examples of gene therapy in skin substitutes are currently active in the areas of innate antimicrobial peptides (e.g., cathelicidins, β-defensins) [67, 68] in allogeneic engineered skin to promote healing of chronic wounds and collagen VII for recessive dystrophic epidermolysis bullosa (RDEB) [69, 70]. These approaches to gene therapies require careful considerations for safety and efficacy in clinical applications. Constitutive overexpression of human beta defensin-3 with a non-viral plasmid DNA in an allogeneic model of a skin substitute has been evaluated for microbial management of contaminated wounds and was not tumorigenic [71]. These kinds of approaches provide novel examples for wound management and correction of congenital skin diseases and open countless opportunities for future reductions of morbidity and mortality from skin wounds. The CRISPR/Cas9 system for gene editing [72] offers an alternative for genetic modification of cells without the associated risks of viral vectors [73, 74].

In addition to unique compositions of cells, gene expression, and scaffolds to construct analogs of skin, a critical and limiting factor to greater availability of skin substitutes is manual fabrication of these complex materials. To address this limitation, numerous methods for robotic fabrication of skin and other tissue substitutes have been described [75]. Many of these approaches are highly precise and involve extrusion of cell-populated matrices into specific shapes for transplantation. For skin models, techniques include multi-layering of multiple cell types [76], “ink-jet printing” [77], or transfer of cell-matrix droplets onto a culture substrate by actuation of a laser pulse [78]. Although these robotic systems accomplish physical transfers with relatively high efficiency, they may injure cells by transient exposures to high pressure, temperature, or chemical toxicities. Importantly, cells suspended in viscous scaffolds may be deprived of cellular attachments to cell surface receptors (e.g., integrins, cadherins), resulting in irreversible proliferative arrest and apoptosis [79]. Avoidance of these kinds of growth inhibitions will be essential to the eventual success of robotic systems. It is important to recognize that these kinds of attachment and signaling deprivations do not occur during fetal morphogenesis or wound healing. Therefore, providing tissue-specific ligands for cell surface receptors, or maintaining signaling pathways that regulate proliferation, will likely be required to optimize the mitotic rates of cells in engineered tissues. One approach to satisfying this requirement is formation of cellular organoids [75] which provide cell-cell attachments to preserve cell cycle signaling without attachment of cells to scaffolds or plastic vessels.

Assessments of skin wounds have progressed from subjective examinations by clinicians to more objective measures with non-invasive instruments for both diagnostic and prognostic evaluations. For diagnostic purposes, scanning laser Doppler flowmetry has been shown to provide accurate assessments of burn depth and color with simultaneous image capture [80–82]. Accuracy in determining the TBSA of burns has also been improved with computer software for digital mapping of skin injuries to better calculate critical interventions such as fluid resuscitation. Three-dimensional photography and laser surface scanning [83, 84] provide topographic data that may be coupled with body mapping to generate virtual representations of patients that can be revised during the hospital course to construct a timeline of clinical progress. Non-invasive instruments for assessments of color, shape, surface texture, visco-elastic properties, blood flow, temperature, pH, surface hydration, and water vapor transmission have been adapted from applications in dermatology for more objective determinations of scars [85]. Although these kinds of instruments have high accuracy, they often provide assessments of individual points within fields of wounds or scars which must be considered in sampling plans for data interpretation. Because point measures typically do not represent heterogeneous wounds, data collection at multiple sites is needed to compensate for the subjective selection of individual points to measure within the treatment field. With these kinds of considerations, application of non-invasive instruments for wound assessments has been shown to correct for inter-rater variability in ordinal or observational evaluations of wounds and scars.

### Regulatory environments and requirements

Safety and efficacy of skin substitutes are regulated in the USA by the US Food and Drug Administration (FDA). Biologic skin substitutes have increased in complexity from models that replace either dermis or epidermis, to dermal-epidermal models, to those that deliver combinations of biopolymer scaffolds, multiple cell types, or multiple cell sources, to those that express gene products for prospective improvements in wound healing. This spectrum of unprecedented materials presented questions regarding the regulatory framework within which each model would be evaluated for consideration of permission to market. Traditionally, the FDA has consisted of three centers for evaluation of human therapeutics: the Center for Devices and Radiologic Health (CDRH), the Center for Biologics Evaluation and Research (CBER), and the Center for Drug Evaluation and Research (CDER). Availability of cadaveric allograft has been provided under regulations for tissue banking, which are administered by CBER. As the spectrum of research models of skin substitutes broadened during the 1980s and 1990s, several investigative therapies had components that required consideration by multiple centers at FDA. The agency responded proactively with two initiatives that have contributed to greater clarity of the regulatory process and with Guidance for Industry [86, 87] on how to propose a path to market.

An early initiative was FDA’s participation in establishment of definitions and standards for tissue-engineered medical products (TEMPs) through formation of a Division IV of Committee F04 for medical devices through the American Society for Testing and Materials (ASTM) [88]. Beginning in 1997, this organization has had members from academics, government, and industry participating in a consensus process for composing definitions of materials and provision of methods for calibration and testing of the materials. With regard to skin substitutes, the ASTM process has resulted in a Standard Guide for Classification of Therapeutic Skin Substitutes [89], providing consensus definitions and nomenclature. The second initiative was FDA’s establishment in 2002 of the Office of Combination Products, by which investigative therapies are reviewed initially for their primary mode of action [90]. This office confers with the Centers for Human Therapeutics to designate new therapies at a lead center at FDA with participation from other centers as appropriate. Together, these initiatives have added clarity to the assignment of novel therapeutics to a designated regulatory path [91]. In addition to providing a framework for innovative investigative therapies, FDA provides “expanded access” or “compassionate use” permissions for treatment of selected conditions that present high risks of mortality or morbidity to patients [92, 93].

More recently, the 21st Century Cures Act (Cures Act) was signed into law in the USA in December, 2016. As the name implies, this law is intended to facilitate and expedite the availability of novel therapies to patients with serious, or potentially life-threatening, conditions. The Cures Act provides for expedited therapeutic development programs including the Regenerative Medicine Advanced Therapy (RMAT) designation for eligible biologics products, and the Breakthrough Devices program which is designed to facilitate the review of certain innovative medical devices [94]. These new designations by FDA are in addition to previous expedited regulatory pathways of Fast Track development [95], Breakthrough Therapy designation [96], Accelerated Approval [97], and Priority Review designation for drugs [98]. Together, these alternative pathways to provisional or full marketing are likely to increase access to the most advanced therapies by patient populations with the greatest medical needs.

## Conclusions

Future prospects for biologic skin substitutes are extensive and diverse. Advances in use and regulation of stem cells in the skin are highly likely to lead to autologous skin substitutes with greater homology to uninjured skin by providing restoration of skin pigmentation, epidermal appendages (hair, sebaceous and sweat glands), a vascular plexus, and subcutaneous tissues. Genetic modification of autologous cells opens tremendous opportunities for regulation of wound closure, reductions in scar formation, and correction of congenital diseases. As these advances in biologic skin substitutes translate into clinical care, it can be predicted with confidence that reductions in morbidity from acquired and congenital skin diseases will also be realized.

## Abbreviations

ASTM: American Society for Testing and Materials
BTM: BioTemporizing Matrix
CBER: Center for Biologics Evaluation and Research
CDER: Center for Drug Evaluation and Research
CDRH: Center for Devices and Radiologic Health
CRISPR: Clustered Regularly Interspaced Short Palindromic Repeats
Cures Act: The 21st Century Cures Act
FDA: Food and Drug Administration
RMAT: Regenerative Medicine Advanced Therapy
STSG: Split-thickness skin graft
TEMPs: Tissue-engineered medical products
RDEB: recessive dystrophic epidermolysis bullosa

## References

[1] Finnerty CC, Capek K, Voigt C, Hundeshagen G, Cambiaso-Daniel J, Porter C, Sousse LE, El Ayadi A, Zapata-Sirvent R, Guillory A (2017). The P50 research center in perioperative sciences: how the investment by the National Institute of General Medical Sciences in Team Science has reduced post-burn mortality. J Trauma Acute Care Surg.

[2] Warden GD, Saffle JR, Kravitz M (1982). A two-stage technique for excision and grafting of burn wounds. J Trauma.

[3] Wood FM, Giles N, Stevenson A, Rea S, Fear M (2012). Characterisation of the cell suspension harvested from the dermal epidermal junction using a ReCell(R) kit. Burns.

[4] Green H, Kehinde O, Thomas J (1979). Growth of human epidermal cells into multiple epithelia suitable for grafting. Proc Natl Acad Sci U S A.

[5] Boyce ST, Hansbrough JF (1988). Biologic attachment, growth, and differentiation of cultured human epidermal keratinocytes on a graftable collagen and chondroitin-6-sulfate substrate. Surgery.

[6] Falanga V, Sabolinski M (1999). A bilayered living skin construct (APLIGRAF) accelerates complete closure of hard-to-heal venous ulcers. Wound Repair Regen.

[7] Griffiths M, Ojeh N, Livingstone R, Price R, Navsaria H (2004). Survival of Apligraf in acute human wounds. Tissue Eng.

[8] Centanni JM, Straseski JA, Wicks A, Hank JA, Rasmussen CA, Lokuta MA, Schurr MJ, Foster KN, Faucher LD, Caruso DM (2011). StrataGraft skin substitute is well-tolerated and is not acutely immunogenic in patients with traumatic wounds: results from a prospective, randomized, controlled dose escalation trial. Ann Surg.

[9] Parenteau NL, Sabolinski M, Prosky S, Nolte C, Oleson M, Kriwet K, Bilbo P (1996). Biological and physical factors influencing the successful engraftment of a cultured human skin substitute. Biotechnol Bioeng.

[10] Alexander JW, MacMillan BG, Law E, Kittur DS (1981). Treatment of severe burns with widely meshed skin autograft, and meshed skin allograft overlay. J Trauma.

[11] Medina A, Riegel T, Nystad D, Tredget EE (2016). Modified meek micrografting technique for wound coverage in extensive burn injuries. J Burn Care Res.

[12] Gallico Iii GG, O'Connor NE, Compton CC, Remensynder JP, Kehinde O, Green H (1989). Cultured epithelial autografts for giant congenital nevi. Plast Reconstr Surg.

[13] Nikkhah D, Booth S, Tay S, Gilbert P, Dheansa B (2015). Comparing outcomes of sheet grafting with 1:1 mesh grafting in patients with thermal burns: a randomized trial. Burns.

[14] Compton CC, Warland G, Kratz G (1998). Melanocytes in cultured epithelial grafts are depleted with serial subcultivation and cryopreservation: implications for clinical outcome. J Burn Care Rehabil.

[15] Harriger MD, Warden GD, Greenhalgh DG, Kagan RJ, Boyce ST (1995). Pigmentation and microanatomy of skin regenerated from composite grafts of cultured cells and biopolymers applied to full-thickness burn wounds. Transplantation.

[16] Greenhalgh DG (2015). A primer on pigmentation. J Burn Care Res.

[17] Heimbach D, Luterman A, Burke J, Cram A, Herndon D, Hunt J, Jordan M, McManus W, Solem L, Warden G, Zawacki B. Artificial dermis for major burns: a multi-center randomized clinical trial. Ann Surg. 1988;208(3):313–9. 10.1097/00000658-198809000-00008 PMC1493652 3048216

[18] Atherton DD, Tang R, Jones I, Jawad M (2010). Early excision and application of matriderm with simultaneous autologous skin grafting in facial burns. Plast Reconstr Surg.

[19] Jansen LA, De CP, Guay NA, Lineaweaver WC, Shokrollahi K (2013). The evidence base for the acellular dermal matrix AlloDerm: a systematic review. Ann Plast Surg.

[20] Gravante G, Delogu D, Giordan N, Morano G, Montone A, Esposito G (2007). The use of Hyalomatrix PA in the treatment of deep partial-thickness burns. J Burn Care Res.

[21] Greenwood JE, Dearman BL (2012). Split skin graft application over an integrating, biodegradable temporizing polymer matrix: immediate and delayed. J Burn Care Res.

[22] Greenwood JE, Wagstaff MJ, Rooke M, Caplash Y (2016). Reconstruction of extensive calvarial exposure after major burn injury in 2 stages using a biodegradable polyurethane matrix. Eplasty.

[23] Muangman P, Deubner H, Honari S, Heimbach DM, Engrav LH, Klein MB, Gibran NS (2006). Correlation of clinical outcome of integra application with microbiologic and pathological biopsies. J Trauma.

[24] Heimbach DM, Warden GD, Luterman A, Jordan MH, Ozobia N, Ryan CM, Voigt DW, Hickerson WL, Saffle JR, DeClement FA (2003). Multicenter postapproval clinical trial of Integra dermal regeneration template for burn treatment. J Burn Care Rehabil.

[25] Branski LK, Herndon DN, Pereira C, Mlcak RP, Celis MM, Lee JO, Sanford AP, Norbury WB, Zhang XJ, Jeschke MG (2007). Longitudinal assessment of Integra in primary burn management: a randomized pediatric clinical trial. Crit Care Med.

[26] Kopp J, Magnus NE, Rubben A, Merk HF, Pallua N (2003). Radical resection of giant congenital melanocyte nevus and reconstruction with meek-graft covered integra dermal template. Dermatol Surg.

[27] Moiemen NS, Vlachou E, Staiano JJ, Thawy Y, Frame JD (2006). Reconstructive surgery with Integra dermal regeneration template: histologic study, clinical evaluation, and current practice. Plast Reconstr Surg.

[28] Herndon DN (2012). Total burn care.

[29] Kagan RJ, Robb EC, Plessinger RT (2005). Human skin banking. Clin Lab Med.

[30] Harris RP, Compton JB, Abstan S (1976). Comparison of fresh, frozen, and lyophilized porcine skin as xenografts on burned patients. Burns.

[31] Burkey B, Davis W, Glat PM (2016). Porcine xenograft treatment of superficial partial-thickness burns in paediatric patients. J Wound Care.

[32] Hermans MH (2014). Porcine xenografts vs. (cryopreserved) allografts in the management of partial thickness burns: is there a clinical difference?. Burns.

[33] Schurr MJ, Foster KN, Lokuta MA, Rasmussen CA, Thomas-Virnig CL, Faucher LD, Caruso DM, len-Hoffmann BL (2012). Clinical evaluation of NIKS-based bioengineered skin substitute tissue in complex skin defects: phase I/IIa clinical trial results. Adv Wound Care (New Rochelle).

[34] Sood R, Roggy D, Zieger M, Balledux J, Chaudhari S, Koumanis DJ, Mir HS, Cohen A, Knipe C, Gabehart K (2010). Cultured epithelial autografts for coverage of large burn wounds in eighty-eight patients: the Indiana University experience. J Burn Care Res.

[35] Gravante G, Di Fede MC, Araco A, Grimaldi M, De AB, Arpino A, Cervelli V, Montone A (2007). A randomized trial comparing ReCell system of epidermal cells delivery versus classic skin grafts for the treatment of deep partial thickness burns. Burns.

[36] Duranceau L, Genest H, Bortoluzzi P, Moulin V, Auger FA, Germain L (2014). Successful grafting of a novel autologous tissue-engineered skin substitutes (dermis and epidermis) on twelve burn patients. J Burn Care Res.

[37] Boyce ST, Warden GD (2002). Principles and practices for treatment of cutaneous wounds with cultured skin substitutes. Am J Surg.

[38] Hansbrough JF, Boyce ST, Cooper ML, Foreman TJ (1989). Burn wound closure with cultured autologous keratinocytes and fibroblasts attached to a collagen-glycosaminoglycan substrate. JAMA.

[39] Boyce ST, Simpson PS, Rieman MT, Warner PM, Yakuboff KP, Kevin BJ, Nelson JK, Fowler LA, Kagan RJ (2017). Randomized, paired-site comparison of autologous engineered skin substitutes and split-thickness skin graft for closure of extensive, full-thickness burns. J Burn Care Res.

[40] Desai MH, Mlakar JM, McCauley RL, Abdullah KM, Rutan RL, Waymack JP, Robson MC, Herndon DN (1991). Lack of long term durability of cultured keratinocyte burn wound coverage: a case report. J Burn Care Rehabil.

[41] Wood FM, Kolybaba ML, Allen P (2006). The use of cultured epithelial autograft in the treatment of major burn injuries: a critical review of the literature. Burns.

[42] Sood R, Roggy DE, Zieger MJ, Nazim M, Hartman BC, Gibbs JT (2015). A comparative study of spray keratinocytes and autologous meshed split-thickness skin graft in the treatment of acute burn injuries. Wounds.

[43] Boyce ST, Medrano EE, Abdel-Malek ZA, Supp AP, Dodick JM, Nordlund JJ, Warden GD (1993). Pigmentation and inhibition of wound contraction by cultured skin substitutes with adult melanocytes after transplantation to athymic mice. J Invest Dermat.

[44] Bottcher-Haberzeth S, Klar AS, Biedermann T, Schiestl C, Meuli-Simmen C, Reichmann E, Meuli M (2013). “Trooping the color”: restoring the original donor skin color by addition of melanocytes to bioengineered skin analogs. Pediatr Surg Intl.

[45] Duval C, Chagnoleau C, Pouradier F, Sextius P, Condom E, Bernerd F (2012). Human skin model containing melanocytes: essential role of keratinocyte growth factor for constitutive pigmentation-functional response to alpha-melanocyte stimulating hormone and forskolin. Tissue Eng Part C Methods.

[46] Supp DM, Wilson-Landy K, Boyce ST (2002). Human dermal microvascular endothelial cells form vascular analogs in cultured skin substitutes after grafting to athymic mice. FASEB J.

[47] Black AF, Berthod F, L'Heureux N, Germain L, Auger FA (1998). In vitro reconstruction of a human capillary-like network in a tissue-engineered skin equivalent. FASEB J.

[48] Tremblay PL, Hudon V, Berthod F, Germain L, Auger FA (2005). Inosculation of tissue-engineered capillaries with the host’s vasculature in a reconstructed skin transplanted on mice. Am J Transplant.

[49] Bhowmick S, Scharnweber D, Koul V (2016). Co-cultivation of keratinocyte-human mesenchymal stem cell (hMSC) on sericin loaded electrospun nanofibrous composite scaffold (cationic gelatin/hyaluronan/chondroitin sulfate) stimulates epithelial differentiation in hMSCs: in vitro study. Biomaterials.

[50] Klar AS, Biedermann T, Michalak K, Michalczyk T, Meuli-Simmen C, Scherberich A, Meuli M, Reichmann E (2017). Human adipose mesenchymal cells inhibit melanocyte differentiation and the pigmentation of human skin via increased expression of TGF-beta1. J Invest Dermatol.

[51] Huang S, Lu G, Wu Y, Jirigala E, Xu Y, Ma K, Fu X (2012). Mesenchymal stem cells delivered in a microsphere-based engineered skin contribute to cutaneous wound healing and sweat gland repair. J Dermatol Sci.

[52] Morissette Martin P, Maux A, Laterreur V, Mayrand D, V LG, Moulin VJ, Fradette J. Enhancing repair of full-thickness excisional wounds in a murine model: impact of tissue-engineered biological dressings featuring human differentiated adipocytes. Acta Biomaterialia. 2015;22:39–49. 10.1016/j.actbio.2015.04.036 25934321

[53] Blais M, Parenteau-Bareil R, Cadau S, Berthod F (2013). Concise review: tissue-engineered skin and nerve regeneration in burn treatment. Stem Cells Transl Med.

[54] Sriwiriyanont P, Lynch KA, McFarland KL, Supp DM, Boyce ST (2013). Characterization of hair follicle development in engineered skin substitutes. PLoS One.

[55] Gagnon V, Larouche D, Parenteau-Bareil R, Gingras M, Germain L, Berthod F (2011). Hair follicles guide nerve migration in vitro and in vivo in tissue-engineered skin. J Invest Dermatol.

[56] Agabalyan NA, Rosin NL, Rahmani W, Biernaskie J (2017). Hair follicle dermal stem cells and skin-derived precursor cells: exciting tools for endogenous and exogenous therapies. Exp Dermatol.

[57] Gledhill K, Guo Z, Umegaki-Arao N, Higgins CA, Itoh M, Christiano AM (2015). Melanin transfer in human 3D skin equivalents generated exclusively from induced pluripotent stem cells. PLoS One.

[58] Itoh M, Umegaki-Arao N, Guo Z, Liu L, Higgins CA, Christiano AM (2013). Generation of 3D skin equivalents fully reconstituted from human induced pluripotent stem cells (iPSCs). PLoS One.

[59] Boyce ST, Lloyd CM, Kleiner MC, Swope VB, Abdel-Malek Z, Supp DM (2017). Restoration of cutaneous pigmentation by transplantation to mice of isogeneic human melanocytes in dermal-epidermal engineered skin substitutes. Pigment Cell Melanoma Res.

[60] Zhang Y, Tomann P, Andl T, Gallant NM, Huelsken J, Jerchow B, Birchmeier W, Paus R, Piccolo S, Mikkola ML (2009). Reciprocal requirements for EDA/EDAR/NF-kappaB and Wnt/beta-catenin signaling pathways in hair follicle induction. Dev Cell.

[61] Morgan BA (2014). The dermal papilla: an instructive niche for epithelial stem and progenitor cells in development and regeneration of the hair follicle. Cold Spring Harb Perspect Med.

[62] Morgan JR, Barrandon Y, Green H, Mulligan RC (1987). Expression of an exogenous growth hormone gene in transplantable human epidermal cells. Science.

[63] Wilson JM, Birinyi LK, Salomon RN, Libby P, Callow AD, Mulligan RC (1989). Genetically modified endothelial cells in the treatment of human diseases. Trans Assoc Am Physicians.

[64] Selden RF, Skoskiewicz MJ, Howie KB, Russell PS, Goodman HM (1987). Implantation of genetically engineered fibroblasts into mice: implications for gene therapy. Science.

[65] Clement F, Grockowiak E, Zylbersztejn F, Fossard G, Gobert S, Maguer-Satta V (2017). Stem cell manipulation, gene therapy and the risk of cancer stem cell emergence. Stem Cell Investig.

[66] White M, Whittaker R, Gandara C, Stoll EA (2017). A guide to approaching regulatory considerations for lentiviral-mediated gene therapies. Hum Gene Ther Methods.

[67] Bals R, Weiner DJ, Moscioni AD, Meegalla RL, Wilson JM (1999). Augmentation of innate host defense by expression of a cathelicidin antimicrobial peptide. Infect Immun.

[68] Comer AR, Rasmussen CA, Thomas-Virnig CL, Lokuta MA, Shaughnessy LM, Schlosser SJ, Johnston CE, Bauer RL, Cleven TD, Wieczorek NC, et al. Preclinical development and planned clinical evaluation of a human skin substitute engineered to secrete elevated levels of a host defense peptide. Proceedings of the Innovations in Wound Healing Conference. 2014; (abstract).

[69] Siprashvili Z, Nguyen NT, Bezchinsky MY, Marinkovich MP, Lane AT, Khavari PA (2010). Long-term type VII collagen restoration to human epidermolysis bullosa skin tissue. Hum Gene Ther.

[70] Ortiz-Urda S, Lin Q, Green CL, Keene DR, Marinkovich MP, Khavari PA (2003). Injection of genetically engineered fibroblasts corrects regenerated human epidermolysis bullosa skin tissue. J Clin Invest.

[71] Gibson AL, Thomas-Virnig CL, Centanni JM, Schlosser SJ, Johnston CE, Van Winkle KF, Szilagyi A, He LK, Shankar R, Allen-Hoffmann BL (2012). Nonviral human beta defensin-3 expression in a bioengineered human skin tissue: a therapeutic alternative for infected wounds. Wound Repair Regen.

[72] Komor AC, Badran AH, Liu DR (2017). CRISPR-based technologies for the manipulation of eukaryotic genomes. Cell.

[73] Go DE, Stottmann RW (2016). The impact of CRISPR/Cas9-based genomic engineering on biomedical research and medicine. Curr Mol Med.

[74] Peng R, Lin G, Li J (2016). Potential pitfalls of CRISPR/Cas9-mediated genome editing. FEBS J.

[75] Patra S, Young V (2016). A review of 3D printing techniques and the future in biofabrication of bioprinted tissue. Cell Biochem Biophys.

[76] Lee V, Singh G, Trasatti JP, Bjornsson C, Xu X, Tran TN, Yoo SS, Dai G, Karande P (2014). Design and fabrication of human skin by three-dimensional bioprinting. Tissue Engineering Part C Methods.

[77] Cui X, Boland T (2009). Human microvasculature fabrication using thermal inkjet printing technology. Biomaterials.

[78] Koch L, Deiwick A, Schlie S, Michael S, Gruene M, Coger V, Zychlinski D, Schambach A, Reimers K, Vogt PM (2012). Skin tissue generation by laser cell printing. Biotechnol Bioeng.

[79] Cho HJ, Youn SW, Cheon SI, Kim TY, Hur J, Zhang SY, Lee SP, Park KW, Lee MM, Choi YS (2005). Regulation of endothelial cell and endothelial progenitor cell survival and vasculogenesis by integrin-linked kinase. Arterioscler Thromb Vasc Biol.

[80] Burke-Smith A, Collier J, Jones I (2015). A comparison of non-invasive imaging modalities: Infrared thermography, spectrophotometric intracutaneous analysis and laser Doppler imaging for the assessment of adult burns. Burns.

[81] Jaskille AD, Ramella-Roman JC, Shupp JW, Jordan MH, Jeng JC (2010). Critical review of burn depth assessment techniques: part II. Review of laser doppler technology. J Burn Care Res.

[82] Ida T, Iwazaki H, Kawaguchi Y, Kawauchi S, Ohkura T, Iwaya K, Tsuda H, Saitoh D, Sato S, Iwai T (2016). Burn depth assessments by photoacoustic imaging and laser Doppler imaging. Wound Repair Regen.

[83] Farrar E, Pujji O, Jeffery S (2017). Three-dimensional wound mapping software compared to expert opinion in determining wound area. Burns.

[84] Bailey JK, Burkes SA, Visscher MO, Whitestone J, Kagan RJ, Yakuboff KP, Warner P, Randall Wickett R (2012). Multimodal quantitative analysis of early pulsed-dye laser treatment of scars at a pediatric burn hospital. Dermatol Surg.

[85] Serup J, Jemed GBE, Grove GL. Handbook of non-invasive methods and the skin, 2nd edn. CRC Press, Inc.: Boca Raton; 2006.

[86] US Food and Drug Administration (1998). Guidance for industry: guidance for human somatic cell therapy and gene therapy. Federal Register.

[87] US Food and Drug Administration (2006). Guidance for industry: chronic cutaneous ulcer and burn wounds - developing products for treatment. Federal Register.

[88] American Society for Testing and Materials (2007). Standard guide for preservation of tissue engineered medical products. In., Report No.: F 2386-04.

[89] American Society for Testing and Materials (2009). Standard guide for classification of therapeutic skin substitutes. In., Report No.: F 2311-08.

[90] US Food and Drug Administration (2011). Guidance for industry: how to write a request for designation.

[91] Witten CM, McFarland RD, Simek SL (2015). Concise review: the U.S. Food and Drug Administration and Regenerative Medicine. Stem Cells Transl Med.

[92] US Food and Drug Administration (2014). Expanded access for medical devices: compassionate use.

[93] US Food and Drug Administration (2017). Expanded access (Compassionate use).

[94] US Food and Drug Administration (2017). 21st century cures act.

[95] US Food and Drug Administration (2017). Fast track.

[96] US Food and Drug Administration (2017). Breakthrough therapy.

[97] US Food and Drug Administration (2017). Accelerated approval.

[98] US Food and Drug Administration (2017). Priority review.

[99] Boyce ST (2001). Design principles for composition and performance of cultured skin substitutes. Burns.

[100] Boyce ST, Supp DM, Albanna MZ, Holmes JH (2016). Biologic skin substitutes. Skin tissue engineering and regenerative medicine.

[101] Lohana P, Hassan S, Watson SB (2014). Integra in burns reconstruction: our experience and report of an unusual immunological reaction. Ann Burns Fire Disasters.

[102] Ellis CV, Kulber DA (2012). Acellular dermal matrices in hand reconstruction. Plast Reconstr Surg.

[103] Halim AS, Khoo TL, Mohd Yussof SJ (2010). Biologic and synthetic skin substitutes: an overview. Indian J Plast Surg.

[104] DeVries HJC, Mekkes JR, Middelkoop E, Hinrichs WLJ, Wildevuur CHR (1993). Dermal substitutes for full-thickness wounds in one-stage grafting model. Wound Repair Regen.

[105] Myers SR, Partha VN, Soranzo C, Price RD, Navsaria HA (2007). Hyalomatrix: a temporary epidermal barrier, hyaluronan delivery, and neodermis induction system for keratinocyte stem cell therapy. Tissue Eng.

[106] Greenwood JE, Dearman BL (2012). Comparison of a sealed, polymer foam biodegradable temporizing matrix against Integra(R) dermal regeneration template in a porcine wound model. J Burn Care Res.

[107] May SR, DeClement F (1981). Skin banking. Part I. Procurement of transplantable cadaveric allograft skin for burn wound coverage. J Burn Care Rehabil.

[108] Griffiths CEM, Nickoloff BJ (1989). Keratinocyte intercellular adhesion molecule-1 (ICAM-1) expression precedes dermal T lymphocyte infiltration in allergic contact dermatitis (Rhus dermatitis). Am J Pathol.

[109] Carlson M, Faria K, Shamis Y, Leman J, Ronfard V, Garlick J (2011). Epidermal stem cells are preserved during commercial-scale manufacture of a bilayered, living cellular construct (Apligraf(R)). Tissue Eng Part A.

[110] Schurr MJ, Foster KN, Centanni JM, Comer AR, Wicks A, Gibson AL, Thomas-Virnig CL, Schlosser SJ, Faucher LD, Lokuta MA (2009). Phase I/II clinical evaluation of StrataGraft: a consistent, pathogen-free human skin substitute. J Trauma.

[111] Frykberg RG, Marston WA, Cardinal M (2015). The incidence of lower-extremity amputation and bone resection in diabetic foot ulcer patients treated with a human fibroblast-derived dermal substitute. Adv Skin Wound Care.

[112] Harding K, Sumner M, Cardinal M (2013). A prospective, multicentre, randomised controlled study of human fibroblast-derived dermal substitute (Dermagraft) in patients with venous leg ulcers. Intnl Wound J.

[113] Gallico Iii GG, O'Connor NE, Compton CC, Kehinde O, Green H (1984). Permanent coverage of large burn wounds with autologous cultured human epithelium. N Engl J Med.

[114] Campanella SD, Rapley P, Ramelet AS (2011). A randomised controlled pilot study comparing Mepitel ® and SurfaSoft ® on paediatric donor sites treated with Recell ®. Burns.

[115] Boa O, Cloutier CB, Genest H, Labbe R, Rodrigue B, Soucy J, Roy M, Arsenault F, Ospina CE, Dube N (2013). Prospective study on the treatment of lower-extremity chronic venous and mixed ulcers using tissue-engineered skin substitute made by the self-assembly approach. Adv Skin Wound Care.

[116] Boyce ST, Kagan RJ, Greenhalgh DG, Warner P, Yakuboff KP, Palmieri T, Warden GD (2006). Cultured skin substitutes reduce requirements for harvesting of skin autograft for closure of excised, full-thickness burns. J Trauma.

[117] Elsholz F, Harteneck C, Muller W, Friedland K (2014). Calcium--a central regulator of keratinocyte differentiation in health and disease. Eur J Dermatol.

[118] Boyce ST, Ham RG (1983). Calcium-regulated differentiation of normal human epidermal keratinocytes in chemically defined clonal culture and serum-free serial culture. J Invest Dermatol.

[119] Rice RH, Durbin-Johnson BP, Ishitsuka Y, Salemi M, Phinney BS, Rocke DM, Roop DR (2016). Proteomic analysis of loricrin knockout mouse epidermis. J Proteome Res.

[120] Wikramanayake TC, Stojadinovic O, Tomic-Canic M (2014). Epidermal Differentiation in Barrier Maintenance and Wound Healing. Adv Wound Care (New Rochelle).

[121] Feingold KR, Elias PM (2014). Role of lipids in the formation and maintenance of the cutaneous permeability barrier. Biochim Biophys Acta.

[122] Ponec M, Weerheim A, Kempenaar J, Mulder A, Gooris GS, Bouwstra J, Mommaas AM (1997). The formation of competent barrier lipids in reconstructed human epidermis requires the presence of vitamin C. J Invest Dermatol.

[123] Haass NK, Smalley KS, Li L, Herlyn M (2005). Adhesion, migration and communication in melanocytes and melanoma. Pigment Cell Res.

[124] Kramer IJ, Kramer IJ (2016). Integrins, cell survival, and cell proliferation. Cell Signalling.

[125] Kippenberger S, Hofmann M, Zoller N, Thaci D, Muller J, Kaufmann R, Bernd A (2010). Ligation of beta4 integrins activates PKB/Akt and ERK1/2 by distinct pathways-relevance of the keratin filament. Biochim Biophys Acta.

[126] Ohyama M, Zheng Y, Paus R, Stenn KS (2009). The mesenchymal component of hair follicle neogenesis: background, methods and molecular characterization. Exp Dermatol.

[127] Chueh SC, Lin SJ, Chen CC, Lei M, Wang LM, Widelitz R, Hughes MW, Jiang TX, Chuong CM (2013). Therapeutic strategy for hair regeneration: hair cycle activation, niche environment modulation, wound-induced follicle neogenesis, and stem cell engineering. Expert Opin Biol Ther.

[128] Kiso M, Tanaka S, Saba R, Matsuda S, Shimizu A, Ohyama M, Okano HJ, Shiroishi T, Okano H, Saga Y (2009). The disruption of Sox21-mediated hair shaft cuticle differentiation causes cyclic alopecia in mice. Proc Natl Acad Sci USA.

[129] Driskell RR, Clavel C, Rendl M, Watt FM (2011). Hair follicle dermal papilla cells at a glance. J Cell Sci.

[130] Xu M, Horrell J, Snitow M, Cui J, Gochnauer H, Syrett CM, Kallish S, Seykora JT, Liu F, Gaillard D (2017). WNT10A mutation causes ectodermal dysplasia by impairing progenitor cell proliferation and KLF4-mediated differentiation. Nat Commun.

[131] Niemann C, Horsley V (2012). Development and homeostasis of the sebaceous gland. Semin Cell Dev Biol.

[132] McNairn AJ, Doucet Y, Demaude J, Brusadelli M, Gordon CB, Uribe-Rivera A, Lambert PF, Bouez C, Breton L, Guasch G (2013). TGFbeta signaling regulates lipogenesis in human sebaceous glands cells. BMC Dermatol.

[133] Lu C, Fuchs E (2014). Sweat gland progenitors in development, homeostasis, and wound repair. Cold Spring Harb Perspect Med.

[134] Zeng B, Xiao X, Li S, Lu H, Lu J, Zhu L, Yu D, Zhao W: Eight mutations of three genes (EDA, EDAR, and WNT10A) identified in seven hypohidrotic ectodermal dysplasia patients. Genes. 2016 7(9); epub. doi: 10.3390/genes7090065. PMID: 27657131. 10.3390/genes7090065 PMC5042395 27657131

[135] Cui CY, Kunisada M, Esibizione D, Douglass EG, Schlessinger D (2009). Analysis of the temporal requirement for eda in hair and sweat gland development. J Invest Dermatol.

[136] Adameyko I, Lallemend F, Aquino JB, Pereira JA, Topilko P, Muller T, Fritz N, Beljajeva A, Mochii M, Liste I (2009). Schwann cell precursors from nerve innervation are a cellular origin of melanocytes in skin. Cell.

[137] Cichorek M, Wachulska M, Stasiewicz A, Tyminska A (2013). Skin melanocytes: biology and development. Postepy Dermatol Alergol.

[138] Aoki Y, Saint-Germain N, Gyda M, Magner-Fink E, Lee YH, Credidio C, Saint-Jeannet JP (2003). Sox10 regulates the development of neural crest-derived melanocytes in Xenopus. Dev Biol.

[139] Kaucka M, Adameyko I (2014). Non-canonical functions of the peripheral nerve. Exp Cell Res.

[140] Biedermann T, Klar AS, Bottcher-Haberzeth S, Schiestl C, Reichmann E, Meuli M (2014). Tissue-engineered dermo-epidermal skin analogs exhibit de novo formation of a near natural neurovascular link 10 weeks after transplantation. Pediatr Surg Int.

[141] Francois M, Koopman P, Beltrame M (2010). SoxF genes: key players in the development of the cardio-vascular system. Int J Biochem Cell Biol.

[142] Wat JJ, Wat MJ (2014). Sox7 in vascular development: review, insights and potential mechanisms. Int J Dev Biol.

[143] Bruderer M, Alini M, Stoddart MJ (2013). Role of HOXA9 and VEZF1 in endothelial biology. J Vasc Res.

[144] Janani C, Ranjitha Kumari BD (2015). PPAR gamma gene - A review. Diabetes Metab Syndr.

[145] Kang S, Kong X, Rosen ED (2014). Adipocyte-specific transgenic and knockout models. Methods Enzymol.

[146] Harms M, Seale P (2013). Brown and beige fat: development, function and therapeutic potential. Nat Med.

